# Editorial: Endocrine aspects of Noonan syndrome and related syndromes, volume II

**DOI:** 10.3389/fendo.2025.1566521

**Published:** 2025-02-18

**Authors:** Laura Mazzanti, Marco Tartaglia, Giorgio Radetti

**Affiliations:** ^1^ Alma Mater Studiorum, University of Bologna, Bologna, Italy; ^2^ Molecular Genetics and Functional Genomics, Ospedale Pediatrico Bambino Gesù, IRCCS, Roma, Italy; ^3^ Marienklinik, Bolzano, Italy

**Keywords:** Noonan syndrome (NS), RASopathies, lipid profile, bone metabolism, puberty, gonadal function, final height

Pediatric endocrinologists are familiar with Noonan syndrome (NS) as the disorder represents one of the most common conditions affecting growth. Indeed, short stature is a major feature characterizing NS and represents a regular reason for consultation. As a syndromic condition, however, NS is characterized by multisystem involvement, and a multidisciplinary approach is required to a proper patient management and care.

The previous Research Topic dedicated to the endocrine aspects of NS and related disorders was primarily focused on the clinical presentation of the disorder, its underlying causes, and growth-related aspects (1). Specifically, Rodriguez et al. (2) explored various aspects of growth hormone (GH) secretion, GH sensitivity at the tissue level, and factors influencing treatment response. Dahlgren et al. (3) examined spontaneous GH secretion and observed altered secretion patterns and partial GH insensitivity. Faienza et al. (4) presented data collected by a multicenter retrospective study conducted across seven Italian pediatric endocrinology centers describing growth progression toward final height and the effects of recombinant human GH (rhGH) treatment in a large cohort of children with NS, comparing treated and untreated subjects. Stagi et al. (5) provided an updated overview of growth patterns and contributing factors—such as GH deficiency, partial GH insensitivity, and altered response to IGF-1—linked to growth failure in NS patients and emphasized the need for personalized treatment plans that consider genotype, along with individualized follow-up and close monitoring during rhGH therapy. Finally, Melis et al. (6) evaluated the prevalence of endocrine disorders in patients affected with NS and clinically related disorders, noting an increased likelihood of thyroid disease and reduced bone mineral density among the former.

In this second volume, the study by Tamburrino et al. focuses on anthropometric details, body composition, and lipid profiles in NS and the closely related RASopathy, Mazzanti Syndrome. Data were initially compared with those of the normal population as well as between the two disorders. Parameters such as height, BMI, total cholesterol, HDL, triglycerides, apolipoprotein levels, fasting glucose, and insulin were evaluated in a cross-sectional and longitudinal study. The main findings suggest an unfavorable lipid profile in NS patients; particularly in patients with pathogenic *PTPN11* variants, who showed a greater involvement of the lipid profile with high prevalence of low lipid levels, compared to the general population. The study also observed that BMI and gender contribute to this correlation. The authors advocate for further research to systematically investigate lipid metabolism dysregulation in RASopathies and confirm previously unrecognized genotype-phenotype correlations affecting the metabolic profile of these disorders.

Butler et al. address diagnostic and management challenges in NS, and Turner and Prader-Willi syndromes, two common genetic disorders in which poor linear growth is associated with similar comorbidities such as cardiovascular, endocrine and fertility issues. The authors highlight delays in diagnosis and treatment due to insufficient information and inefficient communication within healthcare teams. They stress the importance of a multidisciplinary approach for speed up diagnosis, improve treatment, and reach a more effective caregiver education. The paper also discusses issues related to transitioning from pediatric to adult healthcare systems.

Papadopoulou et al. report on the clinical and bone profiles of NS and related RASopathies. They summarize the overlapping clinical features and the different genetic bases of these disorders. Their study highlights the diverse bone tissue and musculoskeletal system involvement in these disorders. Increased serum concentrations of bone resorption markers and normal levels of bone formation markers were observed. They suggest that modulating specific molecules involved in bone remodeling could influence osteogenic outcomes, potentially identifying new pharmaceutical targets.

Patti et al. discuss puberty characteristics, gonadal function, and the role of the Ras-MAPK signaling pathway in fertility. Based on personal and literature-based evidence, they report the occurrence of delayed puberty onset in NS, compared to healthy peers. They also document that male NS patients are at risk of gonadal dysfunction due to both cryptorchidism and developmental factors. The authors emphasize the need for long-term follow-up studies to assess the impact of delayed puberty on adult height, metabolic profiles, and overall well-being.

Puberty in RASopathies was also investigated by Tamburrino et al., who studied the impact of pubertal timing on growth progression and final height. They show that timing of pubertal onset plays a crucial role in determining final height outcome, as the delayed pubertal onset negatively impacts on the amount of height gained during this critical growth period as well as on the final height. Of note, they show that females exhibit a delayed onset of puberty, along with a reduction in peak height velocity (PHV) and spurt gain, compared to the general population. By contrast, males showed only a mild delay in the age at which they reached PHV and a slight reduction in growth spurt gain.

In conclusion, this second Research Topic focused on the endocrine aspects of NS and related syndromes provides an in-depth analysis of challenges related to diagnosis, metabolic and bone alterations, sexual development and fertility, as well as the impact of pubertal timing on final height. By addressing these critical aspects, we aim to offer caregivers a valuable tool for the accurate diagnosis and effective management of patients affected with these disorders.
